# Therapeutic Modulation of Autophagy in Leukaemia and Lymphoma

**DOI:** 10.3390/cells8020103

**Published:** 2019-01-30

**Authors:** Mojgan Djavaheri-Mergny, Sylvie Giuriato, Mario P. Tschan, Magali Humbert

**Affiliations:** 1 INSERM U1218, Université de Bordeaux, 33076 Bordeaux, France; mojgan.mergny@inserm.fr; 2 INSERM U1138, 75006 Paris, France; 3 Université Paris Descartes, Sorbonne Paris Cité, 75006 Paris, France; 4 Université Pierre et Marie Curie, Sorbonne Université, 75006 Paris, France; 5 Cell Biology and Metabolomics Platforms, Gustave Roussy Cancer Campus, 94800 Villejuif, France; 6 TRANSAUTOPHAGY: European Network for Multidisciplinary Research and Translation of Autophagy Knowledge, COST Action CA15138; sylvie.giuriato@inserm.fr (S.G.); mario.tschan@pathology.unibe.ch (M.P.T.); 7 INSERM, UMR1037 CRCT, F-31000 Toulouse, France; 8 Université Toulouse III-Paul Sabatier, UMR1037 CRCT, F-31000 Toulouse, France; 9 CNRS, ERL5294 CRCT, F-31000 Toulouse, France; 10 Department of Pathology, Boston Children’s Hospital and Harvard Medical School, Boston, MA 02115, USA; 11 European Research Initiative on ALK-related malignancies (ERIA), F-31000 Toulouse, France; 12 Institute of Pathology, Division of Experimental Pathology, University of Bern, Murtenstrasse 31, CH-3008 Bern, Switzerland

**Keywords:** macroautophagy, haematopoiesis, leukaemia, lymphomas, therapy response, ageing

## Abstract

Haematopoiesis is a tightly orchestrated process where a pool of hematopoietic stem and progenitor cells (HSPCs) with high self-renewal potential can give rise to both lymphoid and myeloid lineages. The HSPCs pool is reduced with ageing resulting in few HSPC clones maintaining haematopoiesis thereby reducing blood cell diversity, a phenomenon called clonal haematopoiesis. Clonal expansion of HSPCs carrying specific genetic mutations leads to increased risk for haematological malignancies. Therefore, it comes as no surprise that hematopoietic tumours develop in higher frequency in elderly people. Unfortunately, elderly patients with leukaemia or lymphoma still have an unsatisfactory prognosis compared to younger ones highlighting the need to develop more efficient therapies for this group of patients. Growing evidence indicates that macroautophagy (hereafter referred to as autophagy) is essential for health and longevity. This review is focusing on the role of autophagy in normal haematopoiesis as well as in leukaemia and lymphoma development. Attenuated autophagy may support early hematopoietic neoplasia whereas activation of autophagy in later stages of tumour development and in response to a variety of therapies rather triggers a pro-tumoral response. Novel insights into the role of autophagy in haematopoiesis will be discussed in light of designing new autophagy modulating therapies in hematopoietic cancers.

## 1. Introduction

### Macroautophagy—Janus-Faced Role in Cancer

**Figure 1:**
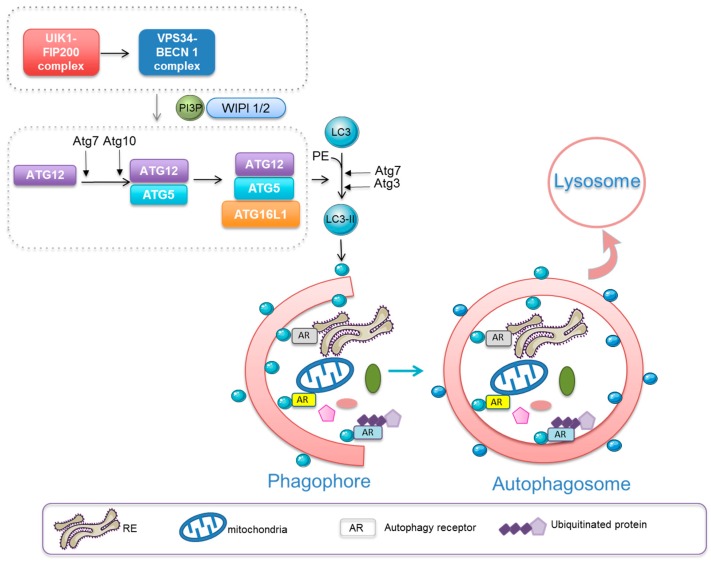
Autophagy occurs through a multistep process. The first step is the formation a double-membrane structure called the phagophore that requires the assistance of two complexes: the ULK1/FIP200 complex and the class III PI3K/BECN1 complex that allows the production of PtIns 3P which bind to WIPI proteins. Subsequently, the autophagosomal membrane expands to sequester cytoplasmic cargoes and to form a vesicle named the autophagosome. This step requires two ubiquitin-like conjugation systems, ATG5-ATG12/ATG16 and ATG8 (LC3-GABARAP)-PE. Apart from the ATG proteins, selective autophagy requires a subset of autophagic adaptors that recognize and bind to specific cargoes (e.g., proteins and mitochondria, endoplasmic reticulum) through ubiquitin-dependent and -independent mechanisms. Autophagy adaptors drive the cargoes to the autophagosomal membrane by binding to LC3/GABARAP-PE through their LIR domain. Finally, the sequestered cargoes are degraded by the lysosomal enzymes upon the fusion of autophagosome with the lysosome.

Macroautophagy (henceforth autophagy) is a vesicular pathway through which cellular components are sequestered into a double membrane vesicle called the autophagosome and then delivered to lysosomes for degradation (Figure 1) [1]. Autophagy is induced in response to a variety of intrinsic and environmental stresses including nutrient and energy limiting conditions, endoplasmic reticulum stress, reactive oxygen species (ROS), hormonal imbalance and exposure to microorganisms [2]. Depending on the stimulus and context, autophagy can sequester bulk cytoplasmic components non-selectively or specific cargoes selectively with the help of autophagy-receptors [3]. Under metabolic demands, autophagy induction generates new precursor components, which are used by cells for energy supply to enable adaptation and cell survival [4]. Autophagy also plays a critical role in the quality control of intracellular components by promoting the removal and replacement of cytotoxic damaged organelles and proteins [5]. Such cellular renewal is especially important in long-lived as well as non-replicative cells such as quiescent and terminally differentiated cells. There is evidence that autophagy plays an essential role in physiological processes that orchestrate development, differentiation, cell survival and immune responses. Defective autophagy is linked to inflammatory and infectious pathologies, cancer, neurodegenerative disorders, liver diseases and ageing [6].

Genetic studies of autophagy in yeast have led to the identification of autophagy (Atg)-related genes whose products drive autophagosome formation through a multistep process [7]. Upstream of the ATG proteins, several signalling pathways regulate autophagy including the mTOR pathway. Inhibition of mTOR leads to activation of the ULK1/Fip200 complex, which in turn promotes the stimulation of the class III PI3K/BECN1 complex. This subsequently leads to the production of PtIns 3P which operates as an initial signal for the formation of a phagophore or initiation membrane. WIPI proteins are PtIns3P binding effectors that allow the recruitment of several multiprotein complexes to the autophagosomal membrane including two ubiquitin-like conjugation systems, ATG5-ATG12-ATG16 and the ATG8 protein family (Microtubule associated protein 1 light chain (LC3)A, B, C, Gamma-aminobutyric acid receptor-associated protein (GABARAP), GABARAP like (GABARAPL) 1, GABARAPL2-phosphatidyl ethanolamine (PE) [8]. These steps are instrumental for the expansion and the closure of the autophagosomal membrane. The last step of autophagy is the degradation of the sequestered cargoes by lysosomal enzymes generating ATP as well as a pool of biomolecules. Selective autophagy requires additional proteins known as autophagic adaptors recruited at the autophagosomal membrane that recognize the cargoes via an LC3-interacting region (LIR) [1]. Several cellular components can be selectively degraded by autophagy for example mitochondria (mitophagy), aggregated proteins (aggrephagy) and the endoplasmatic reticulum (ER-phagy) [9]. Selective autophagy also requires cargo (proteins, mitochondria and pathogens) ubiquitination. In this scenario, autophagy adaptors recognize ubiquitinated cargoes through their ubiquitin binding domain. A subset of autophagy adaptors, including SQSMT1/p62, NBR1, Optineurin and NDP52 operate in selective autophagy through such ubiquitin-dependent mechanisms [10]. Selective autophagy can also occur through an ubiquitin–independent mechanism wherein autophagy adaptors such NIX and FAM134B bind directly to mitochondria and the endoplasmic reticulum (ER), respectively [11,12].

Basal macroautophagy is crucial for maintaining cellular homeostasis in resting cells and also for their proliferation and differentiation [13,14]. In the context of cancer the role of macroautophagy is complex and clearly depends on tumour stage, type and the driving oncogene. In healthy individuals autophagy is regarded as a longevity promoting and tumour suppressing process mainly due to its function to protect cells against genotoxic stress [15]. However, many studies described a tumour-promoting role for macroautophagy once a tumour has formed as well as during metastasis by supporting cancer cell survival. Furthermore, therapy-resistance mechanisms may be based on protective autophagy activated in response to therapy-induced stress [16,17,18,19,20].

In this review, we will summarize the knowledge on autophagy function in healthy hematopoietic cells and the consequences of its deregulation leading to hematopoietic malignancies. In addition, we will give an overview on the effect on autophagy-based pre-clinical therapies in hematopoietic cancers and their potential to improve current therapies. The regulation of autophagy during haematopoiesis has been recently reviewed in Biochemical Pharmacology [21].

## 2. Haematopoiesis Development and Autophagy

### 2.1. Haematopoiesis

Haematopoiesis is a tightly orchestrated physiological process that leads to the generation of all blood cells from a small population of hematopoietic stem cells (HSCs) [22]. HSCs reside in a hypoxic stromal niche within the bone marrow in adults where they maintain their quiescent and self-renewal capacity. HSCs can divide symmetrically, producing two identical daughter HSCs or asymmetrically producing one HSC and a more proliferative cell primed for differentiation. Through these hierarchical events, HSCs produce functionally differentiated progenies, namely myeloid (erythrocytes, megakaryocytes, monocytes, neutrophils, basophils or eosinophils) and lymphoid (T- and B-lymphocytes) cells [23].

**Figure 2:**
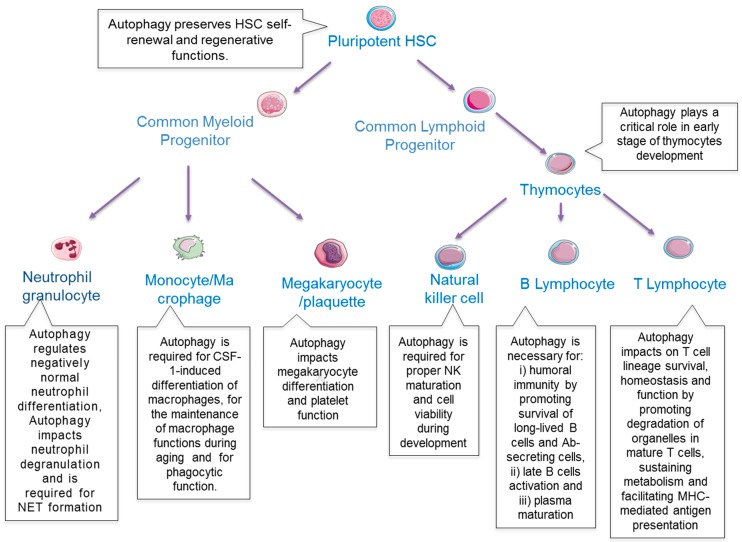
Examples of key functions of autophagy in blood cells.

The self-renewing capability of HSCs allows the lifelong maintenance of an HSC pool. HSCs may acquire mutations leading to a defect in the replenishment of mature blood cells resulting in clonal haematopoiesis or hematopoietic diseases. Therefore, HSCs have developed several adaptive strategies to cope with stressful conditions enabling maintenance of hematopoietic homeostasis [24]. Autophagy is one of the key adaptive mechanisms that occur during haematopoiesis. Here we summarize some recent discoveries that shed new light on the role of autophagy in normal and malignant haematopoiesis (Figure 2).

### 2.2. Autophagy in HSCs

While HSC rarely cycle and are maintained mainly in a quiescent state (G0 phase of the cell cycle) under homeostatic conditions, they can produce billions of mature cells needed in adult human and maintain a pool of HSCs for the entire life of the organism [25,26]. Therefore, HSCs sustain a tight balance between quiescence and active state. Aged HSCs show a reduced potential to regenerate the whole blood system since they are biased towards myelopoiesis [27,28,29,30]. Furthermore, old HSCs show reduced long-term repopulation coupled with reduced chromatin regulation and DNA repair gene expression while genes involved in inflammatory response and stress response are upregulated [31]. Interestingly, while the long-term HSCs population decreased with age, the hematopoietic progenitor cells (HSPCs) compartment is increased leading to clonal haematopoiesis.

Several reports demonstrated a key role of autophagy in HSCs maintenance and function. The conditional deletion of the essential autophagy gene *Atg7* in murine HCSs resulted in accumulation of aberrant mitochondria paralleled by an increase in ROS levels resulting in a drastic increase of DNA damage. Furthermore, the HSC compartment is reduced whereas myeloid progenitors are increased in these mice shifting the differentiation balance towards myelopoiesis [32] similarly to an aged HSC phenotype. Comparable phenotypes were observed when FIP200—a protein of the ULK1/FIP200 complex—was deleted in HSCs, reiterating the role of autophagy in HSCs development [33].

Interestingly, *Atg7* deletion promotes a distinct outcome in HSCs and myeloid cells. In HSCs, *Atg7* deletion promotes irreversible impairment of autophagy and causes death. On the other hand, *Atg7* deficiency in myeloid cells initiates an alternative compensatory autophagy pathway that enables cell viability [34]. This suggests that HCS are more vulnerable to autophagy deficiency than differentiated cells. Indeed, under metabolic stress, long-term HSCs survive by inducing autophagy [34].

Basal levels of autophagy has been shown to control normal HSC differentiation potentially through a mechanism that involves ROS-mediated degradation of the active form of NOTCH [35,36]. Furthermore, basal level of autophagy is essential for removing activated mitochondria and controlling the metabolism of young and old HSC which ultimately preserve HSC self-renewal capacity and regenerative potential [37]. Autophagy was also activated when HSCs were subjected to metabolic stress. Under this condition, autophagy enables cell survival through a mechanism that relies on a FOXO-3-driven pro-autophagy gene program [34]. Hence, the fine-tuned regulation of basal and enhanced levels of autophagy is necessary for proper function and survival of HSCs.

Together, HSCs with impaired autophagy are more prone to ageing leading to increased risk of developing hematopoietic malignancies. Therefore, further studies on autophagy and aging are needed to develop novel strategies to prevent premature aging of HSC.

### 2.3. Autophagy in Development and Differentiation of Lymphocytes

Lymphocytes are comprised of T-, B- and the natural killer cells (NK). T- and B-cells are the major cellular components of the adaptive immune response [38,39].

#### 2.3.1. T Lymphocytes

T cells develop from self-renewing bone marrow HSC. Upon entering the thymus, multipotent progenitors develop towards T-cells and loose self-renewal capacity [40]. During thymic differentiation in mice thymocytes progress from double negative (DN, CD4 CD8) to double positive (DP, CD4+CD8+) stages. A first critical checkpoint in the thymus takes place at the DN3 stage, marked by the rearrangement of the *TCRβ* gene. Following successful rearrangement, the β chain pairs with an invariant pTα chain to form the pre-TCR that drives cell survival, proliferation and differentiation through the DN4 to the DP stages. At this point, successful rearrangement of the TCRα gene allows for the pairing of the α/β chains to produce a functional TCR. Mature single positive T lymphocytes are then released into the periphery. Thus, the recombinases (Rag1/2) that rearrange TCR genes are active at the DN3 and DP stages.

Experiments in chimeric mice generated by transplantation of *Atg5* or *Atg7* knockout foetal liver cells into lethally irradiated congenic host demonstrated that mice with impaired autophagy show normal T cell development but cannot fully reconstitute the lymphoid compartment due to a drastic increase in cell death in the peripheral compartment [41,42]. Furthermore, while expressing normal TCR levels, *Atg5*^−/−^ T cells failed to undergo efficient proliferation after TCR stimulation [41] potentially due to an increase in mitochondria mass [42]. TCR stimulation led to activation of autophagy [43] that restrained activation of a ligand-initiated signalling cascade by inhibiting the NF-κB signalling pathway [44].

Autophagy plays a dual role in T cell subsets depending on the stage of differentiation. Even though autophagy is critical for mature CD4^+^ T cell death after growth factor withdrawal or in T cells lacking FADD activity, caspase 8 or Irgm-1 [45,46], it is linked to survival in other subsets. In T helper 9 cells, autophagy is required for selective degradation of PU-1 which represses differentiation and anti-tumour activity of these cells [47]. In addition, autophagy has also been shown to be critical for the survival integrity of regulatory T cells and the maintenance of long-lived memory T cells, presumably by facilitating the cell adaptation to changing in metabolic demands [48,49]. Moreover, Zhu et al. showed that TBK-binding protein 1 regulates IL-15-induced autophagy in NKT cells and this response operates as a regulator of NKT cell development and survival [50].

Further studies on the role of autophagy in T cell functions at various developmental stages are still needed to have full comprehension of its role during this process.

#### 2.3.2. B Lymphocytes

B cells undergo differentiation through several stages from pro-B, pre-B and immature B to mature B-cells. Upon an immune response, B cells are activated and then fully differentiate into plasma cells, which secrete antibodies against infectious pathogens or for example cancer cells [51].

Reconstitution of irradiated *Rag*^−/−^ mouse with *Atg5*^−/−^ foetal liver cells demonstrated a reduction in peripheral B cells and reduced B cell survival during the final stage of differentiation within the bone marrow (pro-B to pre-B transition) [52]. Using a conditional *Atg5* knockout mouse model under the control of CD19 or Mb1 promoter, Miller et al. and Arnold et al. demonstrated that autophagy plays a critical role in humoral immunity through promoting survival of long-lived B cells and Ab-secreting cells but it is dispensable for pre-B cell transition and B-cell activation under B-cell receptor stimulation [52,53]. Therefore, complete and partial inhibition of autophagy has distinct outcomes in B lymphocyte development. Furthermore, autophagy is necessary for the survival of specific memory B cells but not for the initial generation of memory B cells [54,55]. In addition, WIPI-2 dependent non-canonical autophagy is crucial for B cell activation and mitochondria homeostasis [56]. In line with these data, plasma cells maintenance and antibody response have been shown to be regulated by an ATG5 dependent autophagy [57].

#### 2.3.3. NK Cells

NK cells are the major component of the innate immune response and serve as a first line of defence against cells harbouring a variety of perturbations such as malignant transformation or viral infection. They are part of the recently define innate lymphoid cells family. An NK cell progenitor specific *Atg5* knockout mouse model revealed a crucial role of autophagy in differentiation of these cells [58,59]. Furthermore, the proper association of FOXO1 and ATG7 on the phagophores is needed for the appropriate activation and development of functional NK cells in mice [58].

Moreover, NK cells are crucial for tumour immunosurveillance [60]. Thus, a better understanding of the role of autophagy in this particular lymphocyte subset is crucial for future immunotherapy targeting cancer cells. Indeed, inhibition of the autophagy gene BECN1 in solid tumours induced a massive NK cells infiltration leading to tumour growth inhibition [61,62].

### 2.4. Autophagy in Development and Differentiation of Erythrocytes

During erythropoiesis, cells undergo substantial ultrastructural changes including the removal of nuclei and other intra-cellular organelles. Therefore, it did not come as a surprise that autophagy is critical in this process. Earlier studies suggested that autophagy is involved in the elimination of mitochondria during terminal differentiation of embryonic erythrocytes [63]. Accordingly, the abundance of autophagy vesicles and multi-vesicular bodies has been observed in K562 erythroid cells that underwent differentiation upon hemin treatment [64]. This induction of autophagy relies on the master regulator of haematopoiesis, GATA-1 which has been shown to directly activates gene involved in autophagy [65].

While inhibiting autophagy in K562 cells by knocking out ATG7 or treatment with Bafilomycin A1 led to a reduction of α-globin and γ-globin, the opposite was found when autophagy was activated in K562 cells using rapamycin, starvation or in *Atg7* knockout mouse model [66]. Earlier studies suggested that autophagy is involved in the elimination of mitochondria, referred to as mitophagy, during terminal differentiation of embryonic erythroid cells. NIX (or BNIP3), a BH3-only family member is upregulated during erythroid differentiation [67]. Interestingly, NIX is required for sequestration of mitochondria into the autophagosome during terminal erythroid differentiation [68,69] and interacts directly with GABARAP [70]. Furthermore, during maturation of erythrocytes, autophagosome maturation is dependent on ATG4 [71].

The importance of autophagy during erythropoiesis is further highlighted by several autophagy knockout mouse models. Indeed *Atg7*^−/−^, *Ulk1*^−/−^ and *Nix*^−/−^ murine erythrocytes are not able to degrade mitochondria [32,72,73]. Interestingly, only *Ulk1*^−/−^ erythrocytes have impaired ribosomal clearance suggesting that several distinct pathways are involved in selective degradation of organelles by autophagy during maturation of erythrocytes. One mechanism proposed for the regulation of mitophagy during erythroid differentiation is the requirement of ATG13 to unwanted mitochondria following binding of ULK1 to Hsp90-Cdc37 chaperone complex [74].

Erythropoiesis is aslo regulated by the neutral sphingomyelinase/ceramide axis. The activation of this pathway leads to myelopoiesis through a mechanism that involves the inhibition of autophagy and the modulation of the hematopoietic transcription factors (TFs) GATA-1, GATA-2 and PU.1 [75]. Moreover, NCOA4, a cargo receptor involved in the autophagic turnover of ferritin plays an essential role during erythroid differentiation as evidenced by defective erythropoiesis in NCO4A K562 knockdown cells and a Ncoa4 zebra fish knockout model [76].

### 2.5. Autophagy in Development and Differentiation of Macrophages, Neutrophils and Megakaryocytes

During haematopoiesis, HSCs give rise to multi-potent progenitors (MPPs) that subsequently generate intermediate lineage restricted progenitors. In myeloid differentiation, common myeloid progenitors (CMP) give rise to granulocyte-macrophage (GM) and megakaryocyte-erythroid (MkE) progenitors that subsequently develop in differentiated myeloid cells.

#### 2.5.1. Macrophages

Unstimulated monocytes in circulation are short living cells. Stimuli that induce monocyte-macrophage differentiation induce structural changes and impair the apoptotic program of monocytes [77,78]. Macrophages are key players in innate immune responses to acute and chronic inflammation. During monocyte-macrophage transition induced by GM-CSF, an autophagic survival program is activated [79,80]. Interestingly, M-CSF induced differentiation led to an activation of autophagy but inhibiting key ATG genes did not affect the survival of these cells. These data were confirmed in an *Atg7* knockout mouse model [79]. While GM-CSF stimulation resulted in activation of the MAPK8/JNK1 pathway, M-CSF activates a CAMKK2-PRKAA1 pathway [79,80]. Therefore, further studies are necessary to clarify the interplay between the different kinase cascades, which lead to autophagy activation during macrophage development. Moreover, inhibition of autophagy impairs the ability of mature monocytes to phagocyte bacteria suggesting a role for autophagy in phagocytic function. The activation of autophagy during macrophagic differentiation of monocytes relies on the activation of CAMKK2-PRKAA1-ULK1 pathway and the purinergic receptor P2RY6 [81]. The role of P2RY6 in autophagy regulation was further supported by data showing that the P2RY6 ligand UDP and the specific P2RY6 agonist MRS2693 can restore normal monocyte differentiation through reactivation of autophagy in primary myeloid cells of chronic myelomonocytic leukaemia (CMML) patients. Apart from its role in macrophagic differentiation, autophagy is implicated in the maintenance of macrophage functions during aging. Indeed, deficiency of autophagy due to the loss of *Atg7* gene causes phenotypes similar to aged macrophages including reduced macrophage functions (i.e., phagocytosis and nitrite burst) and pro-inflammatory responses [82]. Therefore, autophagy regulates macrophage homeostasis and function which have potential relevance for the prevention of inflammatory diseases, which progressively increase with age.

Importantly, autophagy is also influencing the alternative activation of macrophages (M2) [83]. Inhibition of autophagy led to a pro-M2 like polarization while activation of autophagy results in impaired alternative activation of macrophages. Interestingly, tumour associated macrophages (TAM) more frequently represent the M2 subtype. Together, deciphering the role of autophagy during macrophage differentiation and activation will contribute to a better understanding of myeloid leukaemia development and may provide new strategies to target TAMs in cancer.

#### 2.5.2. Neutrophils

Neutrophils are the most abundant cell type of the innate immune response. After stimulation, neutrophils can for example degranulate or release chromatin, nuclear histone protein and serine proteases to form neutrophil extracellular traps (NETs) [84]. Unbalanced NETs formation has been linked to autoimmune pathogenesis and inflammatory disorder [85]. The use of 3-Methyladenine or Ammonium Chloride (NH4CL) as inhibitor of autophagy led to increased NET formation in neutrophils [86] suggesting that autophagy may play a protective role in NET formation although these data should be confirmed using more specific autophagy inhibitors. Furthermore, Remijsen Q et al. showed that both autophagy and superoxide generation are required for NET cell death [87]. In the same vein, Kajiume et al. recently demonstrated that human neutrophils undergo autophagic cell death rather than apoptosis [88].

During granulopoiesis, characterized by development from myeloblasts (MB), to promyelocytes (PM), myelocytes (MC), metamyelocytes (MM), band cells (BC) and finally segmented granulocytes (PMN) cells acquire specific morphologic features and generate granules [89]. Interestingly, during these different stages autophagy flux first decreases (MB to MM) to then significantly increase (BC to PMN) sustaining the metabolic reprogramming during neutrophil differentiation [90].

As macrophages, neutrophils can support tumorigenesis and metastasis formation [91]. For example, exosomes secreted by gastric cancer cells cause activation of autophagy in neutrophils by activating the NF-κB pathway through HMGB1/TLR4 [92]. Similarly, patients with systemic sclerosis show increased neutrophil levels with activated autophagy due to the release of platelet-derived microparticles enriched in HMGB1. Thus, autophagic neutrophils demonstrated an enhanced mobilization of their granules, proteolytic activity, prolonged survival and NETs formation [87,93,94,95,96].

Mouse neutrophils deficient for ATG7 or ATG5 are impaired in degranulation of primary and secondary granules [97]. Interestingly, while autophagy-deficient neutrophils circulate more and are more prone towards recruitment to the site of inflammation, they have reduced effector functions. In line with this observation, it has been shown that neutrophils secrete IL-1β via autophagy mediating inflammation [98].

Together, the importance of autophagy during neutrophil development and function highlights its relevance in both immunity and cancer development.

#### 2.5.3. Megakaryocytes

Megakaryocytes (MK) are precursors of platelets that are formed from MK cytoplasm. During maturation, megakaryocytes undergo endomitosis and therefore become polyploid. Then, they extend long branches into blood vessels, also known as proplatelets that upon fission become platelets [99]. In *Atg7*^−/−^ mice megakaryocyte differentiation is impaired due to increased apoptosis and decreased polyploidy [100].

In mouse models, depletion of *Atg7* or *Becn1* resulted in lower platelet numbers with increased size in the peripheral blood [100,101]. In addition, *Atg7*^−/−^ and *Atg7*^+/−^ platelets activation and aggregation were decreased compared to wild type platelets [100]. Accordingly, *Becn1*^+/−^ mice demonstrated an increased bleeding time and aggregation [101]. These findings were confirmed in human cells [101].

## 3. Autophagy and Lymphoid Tumours

### 3.1. Aberrant Autophagy in Lymphomas and Lymphoid Leukaemia

#### 3.1.1. Lymphomas

*Diffuse large B-cell lymphoma* (*DLBCL*) accounts for 33% of B-Non-Hodgkin Lymphoma and is the most common subtype. It corresponds to the malignant counterpart of germinal centre (GC) and post-GC activated cells and is characterized by a diffuse proliferation of large cells harbouring a high mitotic rate. This is an aggressive lymphoma, which arises de novo or is the result of the clinical evolution of less aggressive B-NHL types (like FL and CLL). DLBCL have been subdivided in three major molecular entities based on gene expression analysis [102]: (a) the germinal centre B-cell like derived (GCB) DLBCL, (b) the activated B-cell like (ABC) DLBCL which are related to BCR-activated B cells or B cells committed to plasma cell differentiation and (c) primary mediastinal B cell lymphoma (PMBCL), which arise from post-GC thymic B cells. GCB and ABC subtypes often occur in older male adults (median age: 64 years) whereas PMBCL develops in younger adult women. The GCB subtype is mainly characterized by BCL-2 overexpression, the ABC subtype by NF-κB constitutive activation and BCL-6 overexpression and the PMBCL subtype by amplification of genes involved in T cells immunomodulation. The GCB subtype can be cured by chemoimmunotherapy, whereas more than 50% of the patients presenting the ABC subtype will relapse and die from their malignancy. Recently, the 2016 World Health Organization classification for lymphomas included a new category termed high grade B-cell lymphoma with translocations involving MYC and BCL-2 or BCL-6 [103]. Interestingly, BCL-2 inhibits autophagy by direct binding to BECN1. In line with these observations, patients with a decreased BCL-2 levels have an increase in BECN1 expression that correlates with a favourable clinical outcome [104,105]. In accordance, *Becn1*^+/-^ mice demonstrated a higher frequency of cancer incidence such as lung, liver cancer or B cell lymphoma [106,107]. Another study indicated that the constitutive repression of autophagy responses in BCL-6-driven DLBCL may contribute to lymphomagenesis [108]. On the contrary, Li Y. et al. observed a link between Cullin4B (CUL4B) (a scaffold protein of the CUL4B-RING E3 ubiquitin ligase complex, highly expressed in DLBCL) and autophagy in the positive regulation of DLBCL progression. They showed that CUL4B regulating autophagy occurred through JNK signalling and that the inhibition of proliferation induced by CUL4B deletion may be attributed to the blocking of the pro-survival ability mediated by autophagy [109]. This last study highlights the complexity of autophagy regulation in DLBCL and the necessity to improve our knowledge on the role of autophagy in lymphoma development.

*B-cell Chronic Lymphocytic Leukaemia* (*B-CLL*)/*small lymphocytic lymphoma* (*SLL*) is mainly a disease of older adults (median age: 70 years), characterized by inherent defects in cell death. This leukaemia/lymphoma progresses slowly and, when patients present clinical symptoms affecting their quality of life, they are treated by chemoimmunotherapy. El-Khoury et al. demonstrated that inhibition of autophagy by RNA interference targeting key autophagy genes or by using Chloroquine or 3-Methyladenine in PBMCs from CLL patients decreased cell viability [110] suggesting that B-CLL cells are dependent on autophagy. In line, an IκΒζ mouse model revealed that IκΒζ controls B-lymphocyte proliferation and triggers a Toll-like receptor (TLR)-dependent antibody response [111]. Interestingly, autophagy is required for TLR9-dependent secretion of IgM in IκΒζ positive CLL [112]. In addition, high expression of class 3 PIK3, PIK3R4 and BECN1 are associated with poor outcome in this disease [113].

DAPK1 is an autophagy-associated gene that is frequently silenced in tumours [114]. Interestingly, DAPK1 is inactivated in rare CLL cases by mutations leading to increased binding of HOXB7 to the DAPK1 promoter [115]. Gade et al. demonstrated that loss of DAPK1 expression in CLL is due to a dysfunctional CEBP-β/ATF6 pathway. Furthermore, the inhibition of DAPK1 reduces autophagy and promotes CLL cell growth [116]. This last study points again to the dual role of autophagy in cancer which was supported by other reports. For instance, signaling-lymphocytic-activation-molecule-family1 (SLAMF1) expression is associated with favourable prognosis in CLL cells and is lost in patients with an aggressive form of this disease [117]. Bologna et al. demonstrated that SLAMF1 activates autophagy activity by indirectly stabilizing the BECN1-VPS34 complex. Accordingly, SLAMF1 negative cells are less sensitive to autophagy-inducing therapy [117].

*Mantle cell lymphoma* (*MCL*) is an aggressive disease that constitutes about 5% of B-NHL and rather occurs in older adults. The majority of the patients are treated by immunochemotherapy. Relapses are frequently observed and are mainly treated with the proteasome inhibitor, Bortezomib, [118] or the mTOR inhibitors, Temsirolimus/Everolimus. Indeed, since mantle cell lymphoma overexpress cyclin D1, a key protein involved in the G1/S transition phase and regulated by mTOR signalling, this lymphoma was the first hematologic disease in which the therapeutic efficiency of mTOR inhibitors was investigated [119]. However, the prognosis of patients is dismal and MCL is still considered as an incurable disease, in needs for new therapeutics. It is essentially considered as the malignant counterpart of naïve B cells located in the inner mantle zone of secondary follicles. This lymphoma is characterized by the overexpression of cyclin D1 due to the t(11;14)(q13;q32) chromosomal translocation.

MCL patients express high levels of TG2 and NF-κB [120]. Lowering TG2 in MCL cells decreases proliferation and survival rates. Furthermore, TG2 regulates autophagic flux while inhibiting autophagy by silencing ATG5 resulted in undetectable TG2 levels. In this context, ATG5 knockout cells proliferate at a lower rate compared to autophagy-proficient cells.

Phospholipid scramblase 1 (PLSCR1) is a pro-apoptotic gene upregulated upon 9-cis-retinoic acid and Interferon-a treatment in MCL cell lines [121,122]. Interestingly, PLSCR1 inhibits autophagy activity reducing MCL cell viability [123].

On the contrary, the hedgehog (hH) pathway in MCL cells promotes the infiltration of cells to the bone. Inhibiting the hH pathway using LDE225 treatment increased CXCR4 expression levels and ROS leading to enhanced autophagic activity and cell survival [124].

*Burkitt Lymphoma* (*BL*) is an aggressive immature B-cells disease, characterized by *MYC* gene rearrangement, positivity for early B cell markers and a high mitotic rate. This is a rare disease in adults (1–2% of B-NHL), whereas it accounts for 30% of paediatric lymphomas. These patients are treated by chemoimmunotherapy. Relapsed tumours have dismal prognosis.

Using a mouse model for Burkitt lymphoma, Eµ-Myc transgenic mice, Maclean et al. demonstrated that disruption of lysosomal function using Chloroquine prevents lymphomagenesis linking this disease to autophagy [125].

*Multiple Myeloma* (*MM*) is a cancer of plasma B cell, invading the bone marrow and characterized by a high genomic and phenotypic variability [126]. It corresponds to the unrestrained proliferation of fully differentiated B cells, which excessively produce and secrete monoclonal immunoglobulins. Treatment options have evolved from chemotherapy [127,128] to targeted therapies, including the use of proteasome inhibitors [129], immunomodulatory drugs [130] and chaperone protein inhibitors [131]. However, responses are not durable and MM still represents an incurable disease. This disease primarily occurs in elderly individuals (median age: 69 years). A risk loci for predisposition to MM is mapped to intron 6 of *ATG5* on chromosome 6q21 [132,133]. In addition, high expression of BECN1 or LC3 is associated with a favourable outcome in MM [134]. These two observations suggest a disruption of autophagy is critical in MM disease development, while others demonstrate that autophagy activation is necessary to induce MM survival. Indeed, a recent study described the role of myeloid-derived suppressor cells (MDSC) in promoting MM cell survival and proliferation by activating the AMPK pathway. The authors propose that the pro-survival effect of AMPK may be attributed to the induction of autophagy [135]. Gao D. et al. reported also, in bone marrow cells from patients with untreated MM, the high expression of the long non-coding RNA MALAT and of HMGB1, leading to the promotion of autophagy and survival [136].

The anti-apoptotic CHE-1 protein interacts with RNA polymerase II and regulates gene transcription. Its expression correlates with the progression of MM and is required for cell growth and survival. Interestingly, CHE-1 is phosphorylated upon cellular stress and binds to the Redd1 and Deptor promoters where it activates their transcription and consequently attenuates mTORC activity. CHE-1 expression induces autophagy activity by interfering with both mTORC1 and mTORC2 thereby linking autophagy to survival and progression of MM [137]. In line with these results, several studies demonstrated that inhibition of autophagy reduce MM cell survival [138,139]. Surprisingly, other studies pointed towards a pro-cell death role of autophagy in MM [140,141].

*Primary Effusion Lymphoma* (*PEL*) is a very rare B-cell NHL associated with HHV-8/EBV infections and is mostly observed in human immunodeficiency virus (HIV) positive individuals [142]. PEL present clinically, in its classic form, as malignant lymphomatous effusions in body cavities (pleural, peritoneum, pericardium cavities). Extracavitary PEL, characterized by solid mass lesions, has also been reported [143].

In a recent study, Masud Alam et al. demonstrated that the inhibition of lysosomal degradation using Chloroquine in PEL cells induced ER stress and subsequent apoptosis, thus suggesting that autophagy supports cellular survival in PEL [144].

Interestingly, treatment of PEL cells with Epigallocatechin-3-Gallate (EGCG), the major constituent of green tea, led to suppression of HHV8 replication and ROS production that subsequently induces autophagy and apoptosis [145]. This last study demonstrates that the role of autophagy in PEL development needs more clarification.

*Anaplastic Large Cell Lymphoma* (*ALCL*), *ALK* (*Anaplastic Lymphoma Kinase*) *positive* account for 1–3% of adult T-NHL but correspond to 15% of childhood lymphoma [146]. This malignancy is currently treated by chemotherapy (based on anthracyclines) but refractory or relapsed diseases invariably occur in 30% of the patients, regardless of the drugs and doses used [147]. This lymphoma is predominantly driven by the NPM-ALK oncogene, encoded by the t(2;5)(p23;q35) [148]. Since NPM-ALK is a constitutively active tyrosine kinase [149,150], small molecule inhibitors, such as the first in line Crizotinib [151], have been developed over the past 10 years [152,153]. So far, such targeted therapies have been hampered by the acquisition of resistance to the drug [154,155]. Recently, new promising therapeutic modalities have emerged [146,156,157].

The role of autophagy in ALK+ ALCL development has not been studied so far. Using patient derived cell lines, a single study demonstrated that the inhibition of autophagy by either siRNA directed to *ATG7* or Chloroquine alone did not significantly affect cell viability [158].

*Follicular Lymphoma* (*FL*) represents about 20% of B-NHL, placing it as the second most common lymphoma. It arises in older adults (median age: 60 years) and corresponds to the malignant counterpart of normal germinal centre B cells. Tumour cells commonly invade the bone marrow and are characterized (in 85% of the cases) by the overexpression of the anti-apoptotic protein BCL-2, as a result of the t(14;18)(q32;q12) chromosomal translocation. Other mutations, notably in different epigenetic modifiers such as the mixed-lineage leukaemia 2 (MLL2) protein (a histone H3 methylase), have been linked to the development of FL as well. This disease is the most frequent indolent lymphoma. However, it can evolve to DLBCL (in 20–30% of the cases) over time and it largely remains an incurable disease. The treatment of patients has greatly improved since the use of anti-CD20 monoclonal antibody-based therapy (called Rituximab (RTX)) [159], in combination with chemotherapy [160] or as a single therapeutic agent [161]. Recently, the immunological microenvironment has been proposed as an indicator of prognosis [162,163]. Those last years, new therapeutic options have emerged including immunomodulatory drugs, newer monoclonal antibodies, BH3-mimetics and kinase inhibitors. Regarding the autophagy regulation in B-cells, Mc Carthy et al. found that FL samples showed significantly decreased levels of both SQSMT1/p62 and LC3 compared with reactive B-cells, indicative of an increased autophagy activity in FL. This dysregulation of autophagy in human follicular lymphoma was found independent of overexpression of BCL-2 [164]. Interestingly, FL which express high LC3A levels also harboured a high HIF-1α expression, suggesting a link between hypoxia and activation of autophagy in FL [165]. To our knowledge, no study on the role of autophagy in the disease development or sustainment has yet been performed.

#### 3.1.2. Acute Lymphoid Leukaemia

*Acute Lymphoid Leukaemias* (*ALL*) account for 20% of acute leukaemia in adults and is the most common haematological malignancy in children. It is a heterogeneous disease harbouring different genomic abnormalities such as chromosome number or structure abnormalities, DNA copy number alterations and mutations. 80% of ALL arise from B cell precursors mainly (B-ALL) and 20% from thymocytes (T-ALL). Treatments classically involve chemotherapy (glucocorticoid, Vincristine and an anthracycline) but recent advances in genome profiling not only led to a better stratification of the disease but also to the development of new efficient targeted therapy for some specific subtypes [166,167,168,169,170,171]. As a consequence, the clinical outcome, notably of children with ALL, has considerably improved over the last years, with a cure observed in 80% of the cases. Concerning ALL in adults, only 25 to 50% of patients achieve long-term remission. Thus, new therapeutics are still needed to improve the outcome of patients.

Interestingly, a BECN1 splice variant was identified in the ALL cell line 697 with a deletion of exon 11. BECN1 Del-E11 demonstrated a reduction in autophagy induction [172]. Of note, 697 cells treated with Bafilomycin A1 have a reduction in engraftment in NOD/SCID mice. On the contrary, in a paediatric t(1;19) pre-B acute lymphoblastic leukaemia (pre-B ALL) cell line model, induction of autophagy by either starvation or rapamycin leads to the degradation of DNA pold1 and RNA pol potentially inhibiting cell growth [173]. In line with these observations, the use of Torin-2 on pre-B ALL cells inhibited mTOR activity and increased autophagy paralleled by inhibition of cell growth and cell viability [174], pointing toward a tumour suppressor role of autophagy. Clearly, the role of autophagy in lymphoid malignancies is still debated and might be subtype specific. Therefore, further studies are needed to better understand the role of this recycling mechanism in lympho- and leukemogenesis.

### 3.2. Autophagy-Based Treatment Strategies in Lymphomas and Lymphoid Leukaemia

#### 3.2.1. Lymphomas

*Diffuse large B-cell lymphoma* (*DLBCL*): Several studies related to the role of autophagy in DLBCL treatment have been recently published. In 2012, Jia et al. demonstrated that Bortezomib treatment of DLBCL promoted autophagy, which, through the degradation of IkBa, contributed to NF-κB sustained signalling and drug resistance [175]. They conclude that blocking both autophagy and proteasome pathways could have a great potential in killing DLBCL cells. Another group reasoned that since the phosphatidyl-inositol-3-kinase (PI3K)/Akt/mammalian target of rapamycin (mTOR) signalling pathway is often constitutively activated in DLBCL, treatment with the pan-class I PI3K inhibitor NVP-BKM120 could be beneficial in patients. Using different DLBCL cell lines, the authors showed that this compound decreased cell proliferation and induced apoptosis together with cytoprotective autophagy. Indeed, they further demonstrated that combining autophagy pharmacological inhibition (by using Chloroquine, 3-Methyladenine or Bafilomycin A1) with NVP-BKM120 further decreased DLBCL viability [176]. Yuan et al. demonstrated also that Tenovin-6 inhibited cell proliferation and survival of DLBCL by blocking autophagy [177]. SM1044, a newly synthetized antimalarial artemisinin derivative, demonstrated a significant anti-tumour effect on DLBCL cell lines. SM1044 induces autophagy that promotes Survivin degradation followed by apoptotic cell death [178]. Autophagy inhibitors abrogate SM1044-induced cell death. High expression of Survivin is associated with poor prognosis and can be overcome by inducing a selective degradation of Survivin by autophagy.

Li et al. found that the inhibition of the long non-coding RNA MALAT-1, in several DLBCL cell lines, resulted in autophagy activation and to a higher sensitivity to chemotherapy [179].

*B-cell Chronic Lymphocytic Leukaemia* (*B-CLL*)/*Small Lymphocytic Lymphoma* (*SLL*): Han et al. showed in 2008 that B-CLL cells resistance to TRAIL (TNF-related, apoptosis-inducing ligand) involved the induction of cytoprotective autophagy and that the inhibition of autophagy genes (*Becn-1* and *Atg5*) sensitized the leukemic cells to the drug [180]. Along the same lines, Amrein et al. reported in 2011 that B-CLL cells treatment with Dasatinib, a tyrosine kinase inhibitor, induced cytoprotective autophagy (in a p53-dependent pathway) and drug resistance [181]. Finally, in 2012, Kovaleva et al. demonstrated that, independently to any drug treatment, miR-130a mediated autophagy inhibition (at early step in the autophagy process, through *Atg2B* and *Dicer1* gene expression downregulation) drives B-CLL cells to cell death, thus pointing out the constitutive pro-survival role of autophagy in this disease [182]. Interestingly, the nucleoside analogue, 8-Chloro-adenosine that is in phase I-II clinical trial induces in vitro and in vivo autophagy in CLL [183]. Unfortunately, in the latter study, the authors did not investigate the impact of autophagy modulation on treatment response.

A selective HDAC1, 2, 3 and 11 inhibitor, MGCD0103, triggers cell death in primary CLL cells by inhibiting autophagy [110,184]. Indeed combination treatment of Flavopiridol that induces protective autophagy, with MGCD0103 improves the efficacy of the treatment. Accordingly, in primary CLL cells, Tenovin-6, a Sirtuin targeting small molecule, induces cell death by inhibiting late stage of autophagy, thus preventing its protective effect [185,186].

Recently, in a study aimed to understand the role of the bone marrow microenvironment in CLL resistant cells to Vorinostat treatment, Ding et al. found that autophagy in stroma cells fuels CLL cell growth and that its inhibition remarkably decreases stromal protection and overcomes the resistance to Vorinostat in CLL [187].

*Mantle cell lymphoma* (*MCL*): The cell survival-promoting role of autophagy in MCL has been reported in many studies. Zhang et al. found that under stress, the TG2 (transglutaminase 2)- NF-κB -IL6 signalling pathway triggered autophagy to promote cell survival, lymphoma progression and drug resistance [120]. The same group also demonstrated that the CXCR4/SDF-1 signalling pathway led to autophagy activation, acting as a survival mechanism upon MCL dissemination in the bone marrow [188]. Another study by Mastorci et al. has shown that the 9-cis-retinoic acid (RA)/Interferon (IFN)-α combination increased phospholipid scramblase 1 (PLSCR1) expression and led to the inhibition of cytoprotective autophagy through direct interaction and blockade of the ATG12/ATG5 complex. As a consequence, the authors also demonstrated that the combination of RA/IFN-α with chemotherapy or proteasome inhibitor enhanced apoptosis [123]. Another drug, Flavopiridol, a cyclin-dependent kinase inhibitor (CDKI), was also shown to be effective in MCL cells. However, cytoprotective autophagy restrained its tumour suppressing effect. The combination of 17-AAG (Hsp90 inhibitor) with Flavopiridol enhanced tumour cell apoptosis through BECN-1 degradation, ERK inactivation and autophagy suppression [189]. Finally, autophagy mediated degradation of CD74 was shown to restrain the efficiency of anti-CD74 monoclonal antibody Milatuzumab and to protect MCL cells from such immunotherapy [190,191]. Alinari et al. described how the synthetic sphingosine analogue, FTY720, potentiated the Milatuzumab onco-immunosuppressive effects in MCL by blocking the autophagy-lysosome dependent degradation of CD74 [190]. Altogether, these studies clearly highlight the cytoprotective role of autophagy in MCL, thus opening up a new therapeutic strategy, based on autophagy inhibition, to improve MCL patient’s outcome.

Bortezomib induces NOXA stabilization in MCL cells. Interestingly, inhibition of autophagy by either 3-Methyladenine or Orlistat potentiates NOXA stabilization induced by UPR-inhibition leading to a significant cell death increase [192]. Accordingly, RAD001 induces apoptosis in the majority of MCL cells with low cytotoxic effect on normal T and B cells [193]. Interestingly, RAD001 MCL resistant cells have high autophagic activity compared to responding cells that is linked to their ability to escape the treatment. The resistance can be overcome by the use of Chloroquine.

In a subset of haematological cell lines, including myeloid leukaemia, lymphoid leukaemia, T cell lymphoma and mantle cell lymphoma, Nahimana et al. first demonstrated in 2009 that the treatment of the different cell lines with APO866 (an inhibitor of nicotinamide phosphoribosyltransferase (NAMPT), a key enzyme in nicotinamide adenine dinucleotide (NAD) biosynthesis) promotes cell death associated to autophagy induction [194]. A few years later, the same group further confirmed in a broad panel of haematological cancer cell lines (including T-acute lymphoblastic leukaemia (T-ALL), Burkitt lymphoma (BL), acute myeloid leukaemia (AML), multiple myeloma (MM)) and primary cells isolated from patients with AML and B-CLL that autophagy was indeed essential for APO866 cytotoxic effects [195]. Mechanistically and chronologically, autophagy was found to be activated first (as evidenced by LC3 turnover assay, puncta formation and SQSMT1/p62 degradation) and responsible for the degradation of CAT/catalase, a main cellular antioxidant, thus resulting in an increase in ROS levels, delayed caspases activation and subsequent cell death. Indeed, autophagy inhibition (especially through shRNA targeting ATG5, ATG7 but not through shRNA targeting BECN1) or CAT exogenous addition, blocked APO866-killing effects. Thus, this study places non-canonical autophagy as a critical first cellular answer to APO866, that is necessary to drive different haematological cancer cell lines to apoptotic cell death.

*Burkitt Lymphoma* (*BL*): In a model of MYC-induced lymphoma, Amaravadi et al. have shown in 2007 that combining alkylating chemotherapy with autophagy inhibition (using Chloroquine or shRNA targeting ATG5) enhanced cell death and tumour regression [196]. More recently, other demonstrations for a cytoprotective role of autophagy in BL have been brought by the work of Hart et al., linking accumulation of unfolded proteins in the endoplasmic reticulum (ER) and subsequent higher level of unfolded protein response (UPR) to cytoprotective autophagy activation and tumour cell survival [197]. Another study by Ni et al. demonstrated that treatment of BL cells with Gossypol (a natural BCL-2 inhibitor) induced reactive oxygen species (ROS) production and translocation of high mobility group box 1 (HMGB1) from the nucleus to the cytoplasm, which resulted in the activation of protective autophagy [198]. Finally, Zeng et al. reported in 2013 that recombinant human arginase (rhArg) treatment in Daudi and Raji cells induced proliferation arrest, apoptosis and cytoprotective autophagy. They further demonstrated that combining rhArg treatment with pharmacological or molecular autophagy inhibitors resulted in increased PARP cleavage and higher percentage of apoptotic cells [199]. Recently, Métayer et al. and Fan et al. reported the cytoprotective function of autophagy in asparaginase-treated (and not arginase-treated) Burkitt lymphoma cells [200] and in Vismodegib (an inhibitor of Hedgehog signalling pathway)-treated BL cells [201], respectively. Pujals et al. reported also that the resistance to Nutlin-3 (a p53 pathway activator) induced apoptosis, in EBV-positive BL with a latency III phenotype, involved the activation of autophagy and could be overcome by addition of Chloroquine [202].

Fan J. et al. reported in 2013 that treatment with a chimeric anti-human HLA-DR monoclonal antibody (chLym-1) induced autophagy (as evidenced by LC3 turnover assay, puncta formation and SQSMT1/p62 degradation) and that it constituted as a prerequisite to drive cells towards cell death. Indeed, the pharmacological (3-Methyladenine, Ammonium Chloride) or molecular (siRNA targeting Atg5) inhibition of autophagy suppressed the cytotoxic effects (growth inhibition, caspase-dependent apoptosis, antibody dependent cell death (ADCC) and complement-dependent cytotoxicity (CDC)) of chLym-1. Therefore, this study highlighted the potential benefit of chLym-1 combination with autophagy inducers in B-NHL cells [203].

Granato et al. reported that the treatment of BL cells by Quercetin (a bioflavonoid) resulted in PI3K/Akt/mTOR signalling inhibition and autophagy induction, which contributed to mutant c-Myc reduction. Thus, by stimulating the degradative function of autophagy, Quercetin induced a strong cytotoxic effect against Burkitt’s lymphoma [204].

Li et al. reported recently that autophagy cell death occurred upon arsenic trioxide (As2O3) treatment. The 3-Methyladenine compound was found to reverse BECN1 and BCL-2 expression up- and down-regulation, respectively, which resulted in an increase in cell viability through inhibition of autophagy cell death and apoptosis [205]. In another study, Dong et al. have reported in 2013 that the combination of the histone deacetylase inhibitor valproic acid (VPA) with mTOR inhibitor (Temsirolimus) synergistically inhibited BL cell growth in a murine xenografted model, through the activation of autophagic cell death [206]. Along the same line, a recent work by Ono et al. reported the potent action of rapamycin in inducing autophagic cell death in murine subcutaneously xenografted BL cells. Of note, they highlighted the successful specific delivery of liposome-encapsulated rapamycin, through their conjugation with anti-CD19 antibodies [207]. Finally, Turzanski et al. described the occurrence of autophagic cell death in BL cells owing to rituximab treatment [208].

*Multiple Myeloma* (*MM*): Recently, the role of autophagy in MM development and drugs resistance was investigated (for a review: [209]). The initial work of Hoang et al. demonstrated that autophagy blockade (using Chloroquine or 3-Methyladenine treatment) resulted in MM cells death, thus suggesting the pro-survival role of autophagy under basal conditions [138].

A study of Pan et al. in 2011 demonstrated the cytoprotective role of autophagy following DNA-damaging chemotherapy [210]. Indeed, the authors found that Melphalan and Doxorubicin induced autophagy in different MM cell lines, as evidenced by autophagosome accumulation upon electron microscopy quantification and LC3 turnover assay. Furthermore, autophagy inhibition, through the use of pharmacological inhibitors (3-Methyladenine, Hydroxychloroquine) or shRNA targeting *BECN1* or *ATG5* resulted in a significant improvement of the anti-myeloma activity of the chemotherapeutic treatment, both in vitro (increased apoptosis cell death) and in vivo (xenografted tumour growth prevention through apoptosis induction). Along the same line, HMGB1 knockdown in MM cells or in mice led to autophagy inhibition, which was associated with the potentiation of the dexamethasone chemotherapeutic effects, that is, increased apoptosis in vitro and reduced tumour burden in vivo [211].

Another study by Chen et al. in 2014 reported a cytoprotective function of autophagy in MM cells submitted to Bortezomib treatment [212]. In this setting, the authors further demonstrated that the combination of HDAC inhibitors and BH3-mimetics (ABT-737) resulted in an increase in BIM protein levels, which in turn, directly interacted with BECN1 and blocked the induction of autophagy. Thus, the authors conclude that targeting BIM in MM cells, by disabling cytoprotective autophagy, could overcome acquired Bortezomib resistance [212]. Other studies demonstrated the cytoprotective role of autophagy. The HDAC6 genetic knockout or the use of the HDAC6 inhibitor C1A in MM cells were found to induce cell death by blocking the autophagy degradation pathway of malformed proteins [213]. Similarly, Tigecycline (a glycylcycline antibiotic) induced cytoprotective autophagy in MM cell lines and its combined use with chloroquine was found to synergistically impair the tumour growth in a xenograft model of MM [214]. Elaiophylin, a macrolide antibiotic extracted from Streptomycin melanosporus, is a potent autophagy inhibitor [215]. In p53 mutant MM cells, Elaiphylin demonstrated an anti-myeloma activity in vitro and in vivo. Furthermore, Metformin targets GRP78 (glucose-regulated protein 78)-dependent autophagy in MM cells and potentiate the effect of Bortezomib on tumour regression with a significant survival benefit [216]. Carfilzomib is a second-generation proteasome inhibitor that shows a significant decrease in cell viability when combined with Chloroquine [217]. Furthermore, Chloroquine treatment can overcome the carfilzomib resistance in vitro [218]. In this context of resistance to proteasome inhibitors, Lu et al. found that Profilin1 (PFN1, a cytoskeleton protein) could bind to the BECN1 complex, to promote autophagy and to induce Bortezomib resistance in MM [219]. Zhang et al. found that ClC5, a member of the chloride channel family, promoted survival autophagy and chemoresistance in Bortezomib-treated MM cells [220]. In another study, the chemoresistance to Mephalan was found to be associated with an increased autophagy in MM cells. Mechanistically, the authors described that the high expression of the long non-coding RNA Linc00515 and the subsequent direct inhibition of miR-140-5p resulted in the upregulation of ATG14 levels and autophagy activation [221].

The work of Milan et al. identified SQSMT1/p62 as a novel specific anti-myeloma target, conferring resistance to Bortezomib [222]. Indeed, the authors found that proteasome inhibition not only induced increased SQSMT1/p62 expression levels but also facilitated its interaction with ubiquitinated proteins for subsequent autophagic degradation. Therefore, the lentiviral-mediated depletion in SQSMT1/p62 was found to increase cell sensitivity to proteasome inhibition. The authors concluded that the detection of SQSMT1/p62 aggregates at diagnosis could represent a major prognostic factor for MM patient’s intrinsic susceptibility to proteasome inhibitors.

In plasma cells physiopathology, Lamy et al. proposed recently that autophagy might play an ambivalent role, that is, switching from cytoprotective or cytotoxic functions depending on caspase-10 activity. The authors found that caspase-10 inactivation (using Q-AEVD-OPH or shRNA targeting caspase-10) led to the stabilization of the BCLAF1/BCL-2 complex and the unleashed activation of BECN1, responsible for the autophagy process over-activation, culminating in autophagic cell death [140]. Indeed, the molecular inhibition of *BECN1* and *ATG5*, through targeted siRNA, protected the cells against the autophagic cell death induced by caspase-10 inhibition.

Similarly, the work of Ma et al. pointed out the efficiency of combined chemotherapy (Dexamethasome) and proteasome inhibition (PS-341/Bortezomib) in multiple myeloma cell lines [129]. In this setting, one can hypothesize that survival autophagy was activated following proteasome inhibition, as an alternative way to deal with misfolded protein aggregates and that the combination with dexamethasone, by pushing toward excessive autophagy, induced a switch from cytoprotective to cytotoxic autophagy. In 2008, another molecule, called compound A (CpdA), was described by Chen et al. to stabilize p27 and induce caspase-independent cell death by activation of autophagy. Importantly, this drug overcome resistance to chemotherapy as well as to proteasome inhibitor in MM models, again strongly suggesting that killing of cells occurred through excessive CpdA-mediated autophagy activation [223]. Recently, Betulinic acid (BetA) was found to induce either apoptosis or autophagic cell death in MM cells, depending on the protein phosphatase 2A (PP2A) partner. Indeed, the authors found that under normal conditions, BetA induces caspase-3 activation, cleavage of PP2A, inactivation of Akt and subsequent apoptosis. However, under conditions where apoptosis was blocked, the authors found that PP2A interacts with DAPK to induce autophagic cell death in BetA treated cells [141]. Another study reported that the activation of the endoplasmic reticulum stress, by using the drug Tunicamycin, induced autophagy and apoptosis in MM cells, thereby inhibiting proliferation and chemotherapy resistance [224].

Altogether, these numerous studies highlight autophagy as a promising therapeutic target in MM [225,226].

*Primary Effusion Lymphoma* (*PEL*): In 2011, Sommermann et al. described that the treatment of B-NHL cells (including PEL but also DLBCL and lymphoblastoid cell lines) with chemical an NF-κB inhibitor induced cytoprotective autophagy to overcome the GLUT1 transporter sequestration into the cytoplasm and subsequent leading to a defect in glucose uptake and availability. The authors found indeed that the combination of NF-κB inhibitors with the pharmacological inhibition of autophagy, using Chloroquine and 3-Methyladenine, drove lymphoma cells to metabolic crisis and cell death [227]. In line with these data, Granato et al. reported that autophagy activity in PEL cells is mainly pro-survival and inhibits anti-proliferative effects of proteasome inhibitors [228]. More recently, the same group demonstrated that Quercetin, a flavonoid described as a PI3K/AKT/mTOR and STAT3 inhibitor, induces apoptosis and autophagy in PEL cells. Its combination with autophagy inhibitor (Bafilomycin A or siBECN1) was found to improve cell death in these tumour cells [229].

*Anaplastic Large Cell Lymphoma* (*ALCL*), *ALK* (*Anaplastic Lymphoma Kinase*) *positive:* Regarding the role of autophagy in ALK+ALCL therapy, Mitou et al. demonstrated that Crizotinib (the first ALK tyrosine kinase inhibitor (TKI)) treatment induced cytoprotective autophagy and that combining ALK inhibition with autophagy pharmacological or molecular inhibition improved the TKI drug efficiency [158]. In a follow-up study, Torossian et al. further found that combining ALK inactivation and BCL-2 molecular depletion resulted in the potentiation of autophagy and increased cell death (Torossian et al., manuscript in press [230]).

*Follicular lymphoma* (*FL*): Understanding the role of autophagy in FL therapies is at its premise. So far, the work of Brem et al. described that autophagy could be activated as an alternative cell death program upon BH3-mimetic treatment (Obatoclax) in Rituximab (RTX)-resistant cell lines [231]. Similarly, Leseux et al. demonstrated in 2008 that RTX induced mTOR inactivation in FL cells and further showed that the combination of RTX with rapamycin increased the anti-leukemic effect of the drugs, strongly suggesting that enhanced autophagy may trigger FL cell death [232].

#### 3.2.2. Acute Lymphoid Leukaemia

In various haematological cell lines (including ALL, CLL and multiple myeloma cell lines), Laane et al. demonstrated in 2009 [233,234] that Dexamethasone treatment (a member of the glucocorticoide (GC) class of hormones) induced autophagy first (as evidenced by LC3 turnover assay and puncta formation), which was mandatory for the subsequent cell death by apoptosis. Indeed, autophagy inhibition through siRNA targeting BECN1 or through type III phosphatidylinositol 3-kinase (PI3KIII) complex chemical inhibition (using LY294002 and 3-Methyladenine) led to the inhibition of apoptosis. Along the same line, Polak et al. recently showed that the MEK inhibitor Selumetinib enhanced Dexamethasone toxicity in GC-resistant B-ALL cells through the stimulation of autophagy [235]. Different compounds have been found to induce autophagic cell death in B- and T-ALL. This is the case for Idarubicin (a chemotherapeutic drug (anthracycline)) [236] and for the mTORC1 inhibitor (Everolimus), both of which were found to induce cytotoxic autophagy [237], through the enhanced expression of BECN1 [238].

Back in 1997, Jia et al. first reported that TNFα-mediated apoptosis in T-ALL was abrogated upon autophagy inhibition, pointing to an interplay between the two cell death process [239]. The treatment of ALL cell lines with APO866 (an inhibitor of nicotinamide phosphoribosyltransferase (NAMPT), a key enzyme in nicotinamide adenine dinucleotide (NAD) biosynthesis) was found to induce autophagy as a prerequisite for apoptotic cell death [194]. In line with these findings, Jiang et al. demonstrated recently that autophagy inhibition decreased the apoptotic rate of T-ALL Jurkat cells submitted to selenite treatment [240]. Two other studies demonstrated that glucocorticoide (GC)-induced apoptotic cell death involved the initial activation of autophagy. Of note, resistance to GC occurs in 10% of GC-treated B-ALL [241] and mainly involves a deficiency in apoptosis. Thus, treatment with the BCL-2 inhibitor Obatoclax resulted in the resensitization to drug-induced apoptosis and was found to trigger autophagic cell death [242]. Indeed, further studies showed that Obatoclax was able to activate three types of cell death, that is, apoptosis, autophagy and necroptosis in infant ALL [243]. In this context, Bonapace et al. found that autophagy-dependent necroptosis allowed to overcome GC resistance in T-ALL [244]. Finally, a recent gene expression profiling study in GC sensitive and resistant paediatric patients with B-ALL revealed that an altered expression of autophagy-related genes (converging towards autophagy inhibition) might contribute to the resistance phenotype [245].

As in other diseases, autophagy can also promote survival upon therapy. The work of Wallington-Beddoe et al. demonstrated in 2011 that the immunosuppressive FTY720 compound induced a caspase-independent cell death and a concomitant cytoprotective autophagy in B-ALL cells [246]. This synthetic sphingosine analogue has been described as an autophagy inhibitor in mantle cell lymphoma, highlighting the fact that drugs might have diverse effects on different cells [190]. The Akt inhibitor Triciribine was found to induce cytoprotective autophagy since Chloroquine co-treatment increased its cytotoxic effects [247]. More recently, the impairment of NOTCH1-controlled glutaminolysis (through the inhibition of NOTCH1), combined with the inhibition of the activated cytoprotective autophagy was found to have a synergistic anti-leukemic effect in T-ALL [248]. L-asparaginase (L-asp) is one of the mostly used drug for childhood ALL therapy. Poor response to this therapy has been linked to increased risk of relapse and therapy failures [249,250]. L-asp induces mitochondrial injury together with a cytoprotective autophagy to remove damaged mitochondria. Inhibiting autophagy with Chloroquine improve the treatment responses both in vitro and in vivo under these conditions [251].

## 4. Autophagy and Myeloid Tumours

### 4.1. Aberrant Autophagy in Myeloid Dysplastic Syndromes and Myeloid Leukaemia

#### 4.1.1. Myelodysplastic Syndrome (MDS)

*Myelodysplasic Syndrome* (*MDS*) defines a heterogeneous group of hematopoietic diseases [252], divided in five main subgroups in 1982 that is, Refractory Anaemia (RA), Refractory Anaemia with Ring Sideroblasts (RARS), Refractory Anaemia with Excess of Blasts (RAEB), RAEB in transformation (RAEB-t) and Chronic Myelomonocytic Leukaemia (CMML). These pathologies are characterized by bone marrow cells morphology and abnormal cell numbers, peripheral blood cytopenias and propensity for Acute Myeloid Leukaemia (AML) progression in one third of the cases. MDS are clonal diseases, originated from hematopoietic stem cells [253,254], which acquired and accumulated diverse genetic alterations, mainly the chromosomal deletion (del(5q)) and mutations in genes affecting epigenetic control, transcription, RNA splicing or signal transduction pathways [255]. MDS occurs predominantly in the elderly and can be cured by allogeneic hematopoietic cell transplantation (AHCT) [256]. However, only few patients could benefit from this therapeutic strategy because of their advanced age and because of significant comorbidities and mortality associated with this procedure. According to the type of MDS and to the risk stratification, different therapies have emerged during the last two decades. It includes Erythropoiesis-Stimulating Agents (ESA), Hypomethylating Agents (HMA) such as Azanucleosides, Lenalidomide, Immunosuppressive Therapy (IT) or Iron Chelation Therapy (ICT) [257]. In this framework, several recent studies and one clinical trial highlight the potential of autophagy modulation in the treatment of MDS.

Several studies tried to decipher the role of autophagy in MDS development. Interestingly, increased expression of ATG2B and GSKIP due to germline copy number variation, predispose to several myeloid malignancies such as myeloproliferative neoplasm [258]. On the other hand, Park et al. demonstrated that MDS patients harbouring the mutation U2AF35(S35F) have increased *ATG7* pre-mRNA levels that undergo aberrant distal cleavage at polyadenylation site [259]. This results in a decreased of both, ATG7 expression and autophagic activity sensitizing cells to transformation. In line with this data, nucleated red blood cells of high-risk MDS patients have lower LC3B levels and more defective mitochondria. Furthermore, LC3B levels correlated with haemoglobin levels [260]. In addition, ATG3 is expressed at lower level in MDS patients compared to healthy individuals. Furthermore overexpression of ATG3, in the SKM-1 MDS cell line induces an increase of autophagy activity and a caspase dependent cell death at steady state [261].

#### 4.1.2. Acute Myeloid Leukaemia

*Acute Myeloid Leukaemia* (*AML*) is the most common type (80%) of leukaemia in adults and mainly occurs in the elderly. AML is characterized by an arrest in myeloid differentiation and an aberrant cell survival/proliferation of leukaemia blasts with high clinical heterogeneity between individuals. The heterogeneity of this disease renders the treatment a real challenge in the clinic [262] with the exception of acute promyeloicytic leukaemia (APL) harbouring the chromosomal translocation t(15;17) encoding for the oncogenic fusion protein PML-RARA. Although the World Health Organization recently published a new and more precise classification of AML subtypes [263], very little progress has been made in terms of treatment. According to the Swiss national institute for cancer epidemiology research, the relative cumulative five year survival after AML diagnosis improved modestly between 2001–2007 and 2008–2013 from 19.2% to 23.3% in Switzerland [264]. The median age of AML patients is about 70 years [265] and with the increasing number of elderly people in wealthy countries, the prevalence of AML is expected to increase. Therefore, improvements in therapy are urgently needed.

A body of evidence indicates that primary AML blasts show low autophagy gene levels compared to nonleukemic or differentiation undergoing AML cells [266,267]. In the same vein, the autophagy receptor SQSMT1/p62 was shown to be upregulated during the neutrophil differentiation in APL cells. Accordingly, it was reported that miR-17, -20, -93 and -106, which target SQSMT1/p62, are expressed at higher levels in mouse and human hematopoietic blast cells than in neutrophils [268]. It is assumed that the upregulation of SQSMT1/p62 prevents the accumulation of ubiquitinated protein aggregates during terminal differentiation of APL cells and operates, as a pro-survival cellular mechanism in this context [269,270]. Recent evidence also demonstrated that the SQSMT1/p62 is essential for cell growth and the maintenance of mitochondrial integrity of murine myeloid leukaemia. Indeed, loss of SQSMT1/p62 has been shown to impair leukaemia development and mitophagy in this leukaemia type [271].

Several publications support the idea that the role of autophagy in leukaemia development varies depending on the oncogene that can affect the progression in the disease. The RET proto-oncogene is a receptor tyrosine kinase identified recently as an essential kinase in AML development [272]. Interestingly, RET activated pathways lead to a reduction of autophagy and stabilization of leukemogenic drivers such as mutant FLT3. Furthermore, inhibition of RET leads to FLT3 depletion via autophagy. Interestingly, proteasome inhibitors promote FLT3-ITD degradation through autophagy [273]. Conversely, inhibition of FLT3-ITD mutant in AML cells impairs autophagy-dependent proliferation in vitro and in vivo, indicating that FLT3-ITD supports a high level of basal autophagy. This FLT3-ITD dependent autophagy relies on the ATF4 transcription factor expression [274]. In AML cells with mutant NPM1, PML is stabilized within the cytoplasm. PML cytoplasmic localization leads to phosphorylation of AKT that subsequently activates a pro-survival autophagy [275].

In a MLL-ENL mouse model, inhibition of autophagy via knockout of *Atg7* resulted in a modest increase of leukaemia survival free of mice together with a decrease in leukaemia initiating cells (LICs). MLL-ENL *Atg7*^−/−^ LICs demonstrated an increased ROS production, linked to an increase in mitochondria activity and cell death. Accordingly, MLL-ENL *Atg7*^−/−^ blasts in the peripheral blood are decreased due to enhanced apoptosis [276]. Surprisingly, Watson et al. demonstrated that in MLL-ENL autophagy inhibition by either knocking out *ATG7* or *ATG5* leads to more aggressive leukaemia in vivo. Furthermore, they demonstrated that MLL-ENL cells with decreased autophagy activity allow for abnormal mitochondria activity, proliferation and a glycolytic shift [267]. In an AML1-ETO AML mouse model, autophagy inhibits self-renewal potential of LICs [277].

*Mll-Af9* (MA9) AML cells show high autophagic flux compared to normal bone marrow but interestingly disruption of either *Rb1cc1* or *Atg5* does not affect growth or survival of MA9-AML cells in both in vitro and in vivo [278]. Indeed, Liu et al. demonstrated that autophagy activity is needed for the development of the disease but is dispensable for the maintenance of leukaemia in this particular subtype [279].

The H2.0-like homeobox transcription factor (HLX), which is overexpressed in AML is another factor involved in regulating hematopoietic differentiation. It has been reported that HLX upregulation resulted in AMPK activation and increased viability of AML cells possibly via autophagy activation [280].

Together, AML is a highly heterogeneous disease and therefore it is not surprising that autophagy act as either a tumour promotion or suppression mechanism depending on the AML subtype.

#### 4.1.3. Chronic Myeloid Leukaemia

*Chronic myeloid leukaemia* (*CML*) represents 15% of all leukaemias and mainly occurs in adults (median age at diagnosis: 66). CML is characterized by the translocation t(9;22)(q34;q11) resulting in the expression of the BCR-ABL fusion protein, which harbours an unrestrained tyrosine kinase activity. It presents first as an indolent disease, also called the chronic phase (CP), which can last for many years and corresponds to the expansion of myeloid cell progenitors from leukemic stem cells. The loss of terminal differentiation leads to a more aggressive advanced phase (AP), characterized by the accumulation of either myeloid blasts, pre-B lymphoma blasts or T cells blasts in 65%, 35% and 5% of the cases, respectively. This evolution to “blast crisis” happens through the acquisition of additional chromosomal abnormalities including p53 mutations. How BCR-ABL expression leads to leukemogenesis has been widely investigated and many signalling pathways downstream of the oncogenic tyrosine kinase have been found to be dysregulated. Since 2001, the standard front line therapy for patients with newly diagnosed CML is ABL tyrosine kinase inhibitor (Imatinib) treatment. The remarkable success of Imatinib treatment subsequently led to the new area of “targeted therapy” for many other cancer types with a well-defined molecular abnormality. This treatment efficiently controls disease progression unless mutations in the BCR-ABL oncogene (in 40% of the cases) or other resistance mechanisms, for example, BCR-ABL amplification or increased drug efflux, occur. These resistances occur in 15% of the patients within the first 5 years of the treatment and patients are either treated by second generation TKI or by allogenic hematopoietic cell transplantation. For patients who evolved towards a blast crisis, the prognosis remains poor. Thus, while the current ITK therapy successfully allows to control CML progression for the majority of the patients (85%), future therapeutic strategies are currently investigated to eradicate the disease, notably by targeting the leukemic stem cells, which are known to be insensitive to the TKI treatment [281]. In this framework, the role of autophagy has been found controversial, according to the drug treatment and to the downstream activated signalling pathway. Of note, an autophagy-mediated degradation of the fusion BCR-ABL oncoprotein has also been reported, thus highlighting that a threshold of autophagy induction along with BCR-ABL expression levels might control leukemic cell fate upon treatment [282].

It has been further demonstrated that BCR-ABL1 induces autophagy in a MAPK15-dependent manner which potentially leads to cell transformation [283]. On the other hand Sheng et al. described that BCR-ABL inhibits autophagy in a ATF5-dependent manner but only in transformed cells [284].

Interestingly, Lys05, a second generation of autophagy inhibitor, was shown to reduce CML leukaemia stem cell quiescence and to induce maturation of CML cells [285]. Moreover, Lys05 or PIK-III (an inhibitor of the PtIns3P class III) when combined with Tyrosine kinase inhibitor selectively reduced the number of primary CML LSCs suggesting the relevance of this drug combination strategy for killing cancer stem cells in CML patients [285].

Using a culture strategy by keeping cells first at low oxygen concentrations followed by non-restricted O2 supply, Ianniciello et al. demonstrated that K562 CML cells or primary CML CD34+ cells needed autophagy for commitment but not for proliferation [286]. Furthermore, BIM1 expression was proposed to support the progression of CML towards acute phases. Indeed, BIM1 inhibition triggers CCNG2 expression and a drop in clonogenicity linked to a decrease in tumour-suppressive autophagy response [287].

### 4.2. Autophagy-Based Treatment Strategies in Myeloid Dysplastic Syndromes and Myeloid Leukaemia

#### 4.2.1. Myelodysplastic Syndrome (MDS)

A study performed on SKM1 myeloid cells has shown that their resistance to 5-Azacytidine treatment could be circumvented through autophagy activation [288]. Of note, these results account for the basis of a clinical trial using autophagy activators in the treatment of high risk MDS and AML (Clinical trial number: NCT 01210274, Table 1). Accordingly, another recent study performed by the same group on primary CMML myeloid cells, demonstrated that autophagy activation, through the stimulation of the purinergic receptor P2RY6 and its downstream CAMKK2-PRKAA1-ULK1 signalling pathway, was mandatory to restore the normal differentiation of monocytes to macrophages and thus, could represent a new promising therapy for this pathology [81]. Interestingly, in SKM-1 MDS cells, FOXO3A has been described as a positive regulator of autophagy. Silencing FOXO3A leads to a reduction of autophagy and promotes differentiation induced by decitabine [289]. Dubois et al. reported recently that LAMP2 deficiency (and subsequent major defects in Chaperone Mediated Autophagy (CMA)) in Azacytidine-resistant MDS cells resulted in their hypersensitivity to lysosomes and (macro)autophagy inhibitors. Thus, targeting macroautophagy in CMA deficient LAMP2^low^ MDS/AML patients appears as a new therapeutic option [290].

Autophagy precedes apoptotic cell death in CD34^+^ cells isolated from high-risk MDS and in MDS cell lines following nutrient deprivation and inhibition of the NF-κB-activating IK-κB kinase complex using the BAY11-7082 compound. In this framework, it has been shown that a bioenergic failure drives the activation of autophagy and then apoptosis, as assessed by classical and typical cellular features. However, the authors found that cell death occurred even in the context of autophagy or apoptosis blockade, thus highlighting the high plasticity of MDS cells in their cell death modalities [291]. Bortezomib (Velcade) is a proteasome inhibitor widely used to treat cancers including MDS and AML. Interestingly, Fang et al. demonstrated that Bortezomib induced degradation of TRAF6 via autophagy leading to cell death while inhibiting autophagy using 3-Methyladenine leads to increased survival of MDS and AML cells [292].

#### 4.2.2. Acute Myeloid Leukaemia

Standard treatment is based on chemotherapy, which successfully reduces the bulk of leukemic cells but unfortunately spares the malignant stem or progenitor cells, therefore leading to a high propensity for leukaemia relapses. Many studies have shown the induction of autophagy in AML cells exposed to a variety of new drugs. In accordance with the known dual role of autophagy upon anti-cancer therapies in solid tumours, both pro-survival and pro-death functions have been attributed to autophagy in therapeutically challenged AML [293]. Furthermore, in a few studies activation of autophagy primed AML cells towards other forms of cell death [291]. Finally, a switch from cytotoxic to cytoprotective autophagy has been shown to occur in the same AML cell line, depending on the concentration of AZD8055, a specific mTOR kinase inhibitor [294].

Conventional treatment of AML cells with chemotherapy can induce cytoprotective autophagy through HMGB1 release. Indeed, the use of neutralizing antibodies potentiated the chemotherapy-induced cytotoxicity [295]. Autophagy serves a critical role in protecting AML cells from Cytarabine and in the development of Cytarabine resistance [296]. Targeting autophagy permits to overcome Cytarabine resistance not only in AML cells but also in co-culture of AML cells with marrow-derived mesenchymal stromal cells [297]. Furthermore, Cytarabine treatment induces autophagy in leukaemia-initiating cell and autophagy inhibition sensitizes cells to the anti-leukemic effect of Cytarabine supporting again to the contribution of autophagy to Cytarabine resistance [276]. However, low dose of Cytarabine treatment promotes cytotoxic autophagy in U937 AML cells highlighting that the effect of autophagy on cell demise depends at least in part on the dose of anti-cancer therapy used [298].

Several groups have demonstrated that pro-survival autophagy is activated in AML cells submitted to dual mTORC1/mTORC2 inhibitors [294,299]. Along the same line, it was found that inhibition of mTORC1, following L-asparaginase treatment in AML cells, resulted in cytoprotective autophagy [300].

Simonsen et al. also demonstrated that treatment with HDAC inhibitors induced cytoprotective autophagy in AML1-ETO-positive AML cells, at least in part through mTOR inactivation. Furthermore, the authors showed that inhibition of autophagy by either the concomitant use of 3-Methyadenine or Chloroquine or the molecular down-regulation of ATG7 resulted in increased apoptosis [301]. In the same vein, potent repression of autophagy through ATG7 downregulation was associated with mitochondrial homeostasis defects, ROS accumulation, DNA damage and apoptotic cell death in a specific subset of AML cells (Down syndrome) submitted to HDACi, further highlighting the cytoprotective function of autophagy in this context [302].

The combination of Obatoclax, an inhibitor of the anti-apoptotic proteins BCL-2, BCL-xL and Mcl-1 with Sorafenib (a multi-kinase inhibitor) was also shown to induce cytoprotective autophagy in AML cells. Indeed, the pharmacological inhibition of autophagy by using 3-Methyladenine, Chloroquine or Bafilomycin A1, strongly potentiated cell toxicity of the drug combination [303].

Early studies tended to associate the accumulation of autophagosomes in AML cells to the occurrence of cell death. This was the case in the HL60 AML cell line submitted to Morphinone [304] and to AML cells and primary cells from patients submitted to APO866 (a NAD biosynthesis inhibitor) [194,195]. More recently, Ristic et al. demonstrated the activation of an mTOR-dependent cytotoxic autophagy upon Idarubicin treatment [236]. Interestingly, the AMPK agonist GSK621 has been shown to promote autophagy concomitantly to mTOR activation, a process that leads to AML cell killing [305]. Arsenic trioxide treatment of AML cells was also shown to elicit autophagy cell death through a mechanism that is not fully understood [306].

In addition, autophagy is involved in the degradation of certain AML oncoproteins such as PML-RARA and FLT3-ITD [273,307] but not AML1-ETO [301]. These results demonstrate that autophagy can selectivity eliminate oncoproteins through mechanisms that warrant further investigation. All-trans retinoic acid (ATRA), a potent differentiation agent, has been reported to effectively promote clinical remission of APL when combined with anthracyclines or arsenic trioxide (ATO) [308]. Importantly, several groups showed activation of autophagy during ATRA-induced granulocytic differentiation of APL cells [307,309,310,311,312]. This response occurs through WIPI dependent and BECN1 independent mechanism and is associated with increased expression of GATE16, MAP1S, DRAM1, HMGB1 and SQSMT1/p62 [269,310,312,313,314,315,316]. ATRA treatment also induced selective degradation of ubiquitinated proteins through a SQSMT1/p62 dependent mechanism [269]. While molecular and pharmacological inhibition of autophagy attenuated granulocytic differentiation of APL cells, downregulation of BECN1 and SQSMT1/p62 expressions did not affect this process but rather promoted APL cell death. Autophagy was assumed to promote differentiation of APL cells through a mechanism that relies on PML-RARA degradation. Interestingly, recent studies have revealed that non-coding RNAs, that is, the microRNA (miR) miR125B1 and the long noncoding RNA (lncRNA) HOTAIRM1 could negatively or positively regulate the degradation of PML-RARA during the process of ATRA-mediated myeloid cell differentiation, respectively [317,318]. PML-RARA is also cleared upon arsenic trioxide treatment of APL through a mechanism that involves proteasomal and autophagic pathways [319]. The combination of ATRA with Dasatinib also promoted autophagy and the differentiation of leukemic cells [320]. Finally, Goussetis et al. demonstrated that APO treatment of non-APL cells resulted in autophagic cell death through the activation of the MEK/ERK pathway [306]. However, another study has shown that APO inhibited the autophagic flux [321]. Altogether, these studies point to the distinct roles of several autophagy-regulatory pathways in acute myeloid leukaemia cells demise.

### 4.3. Chronic Myeloid Leukaemia

Goussetis et al. first reported the autophagic degradation of the BCR-ABL oncoprotein in CML cells under arsenic trioxide (ATO) treatment in 2012 [282]. Elzinga et al. further confirmed that Imatinib treatment induced also the sequestration of the BCR-ABL oncoprotein in autophagosomes, allowing its subsequent degradation [322]. These results are important since they highlight for the first time that autophagy intensity and/or duration, within a drug treated leukemic cells, could control its fate, from survival to cell death, through the tight regulation of the driven oncogene expression levels.

Carew et al. reported that the treatment of different CML cell lines and primary CML cells expressing wild-type and Imatinib-resistant mutant forms of BCR-ABL, including T315I, with an histone deacetylase inhibitor (suberoylanilide hydroxamic acid (SAHA)) induced autophagy and that its inhibition (by using Chloroquine) potentiated the anti-neoplastic effects of SAHA [323]. Two years later, Bellodi et al. reported a study of noticeable clinical importance which showed that Imatinib treatment of CML cells induced cytoprotective autophagy and that the inhibition of autophagy, by using pharmacological (Chloroquine, Bafilomycin A1) or RNA interference of essential autophagy genes (ATG5, ATG7) potentiated the drug efficiency not only in CML blast crisis cell lines and CML primary cells but also in leukemic stem cells isolated from patients [324,325,326]. In line with these findings, Crowley et al. reported that pharmacological agents with inherent anti-autophagic activity improved the cytotoxicity of imatinib [327]. More recently, Zeng et al. found a synergistic effect when combining the inhibition of Hedgehog signalling, which is important for the malignant stem cell maintenance and the inhibition of autophagy [328]. Compounds such as Resveratrol, a natural phytoalexin isolated from grapes) and Acadesine (AICAR, 5-aminoimidazole-4-carboxyamide ribonucleoside, known to activate AMPK) were found to induce autophagic cell death in both sensitive or resistant CML cell lines to Imatinib, through AMPK activation and SQSMT1/p62 upregulation [329,330] and through PKC activation [331], respectively. More recently, Tong et al. found that an oncolytic adenovirus expressing BECN1 was efficient in inducing autophagic cell death in different leukemic cell lines and primary leukemic blasts, along with Imatinib resistant cells [332].

## 5. Conclusions

While a lot of effort was put into understanding the role of autophagy in cell transformation and the benefit of autophagy-based treatment strategies, the function of autophagy remains ambiguous in hematopoietic cancers. Furthermore, in many settings autophagy has been shown to play a divergent role in the outcome of hematopoietic cancers, thus, caution should be taken when the modulation of autophagy is considered as a therapeutic strategy to improve the treatment of hematopoietic cancers. Several points have been discussed already to try to solve issues encountered in many different diseases where autophagy-based therapy might be an option [333]. Nevertheless, as shown in Table 1, modulating autophagy in hematopoietic cancer is attractive and may open new avenues for efficient treatment strategies.

In addition, autophagy can be either selective or non-selective and several examples highlight non-canonical autophagy regulation in some hematopoietic cancers. Understanding how the autophagy machinery is involved in healthy and malignant hematopoietic cells as well as identifying the role of specific autophagic cargos during transformation, maintenance and treatment response will help us to have better understanding the role of autophagy in haematopoiesis and to develop new targeted strategies to improve treatment of hematopoietic diseases. Furthermore, recently a study on Chloroquine demonstrated that unlike Bafilomycin A1, Chloroquine and Hydroxychloroquine leads to an accumulation of autophagosomes [334]. Additionally, Chloroquine alters the Golgi organization and causes the vacuolization of lysosomes. Therefore, pre-clinical and clinical studies that used Chloroquine as only autophagy inhibitor should be validated by using more specific autophagy modulators.

## Figures and Tables

**Table 1:** List of clinical trial involving autophagy modulation in hematopoietic cancers.

Haematological Malignancies	Therapeutic Modulation of Autophagy Single Drug or Combination		Clinical Trials	
			Number	Phase
Chronic lymphocytic leukaemia	**Autophagy Inhibition**	Hydroxychloroquine (HCQ)	NCT00771056	II
Multiple myeloma		HCQ + Bortezomib	NCT00568880	I/II
		HCQ + Cyclophosphamide + Dexamethasone + Rapamycin	NCT01689987	I
Lymphoma		Vinblastine	NCT00059839	III
Chronic myeloid leukaemia		HCQ + Imatinib	NCT01227135	II
Acute myelogenous leukaemia		HCQ + Mitoxantone + Etoposide	NCT02631252	I
Chronic myeloid leukaemia	**Autophagy Induction**	Everolimus (RAD001, mTORC1 inhibitor)	NCT01188889	I/II
Chronic lymphocytic leukaemia		Everolimus + Alemtuzumab	NCT00935792	I/II
		CAL-101 (PI3Kd inhibitor)	NCT01539512	III
		Perfosine	NCT00873457	II
Relapsed follicular or mantle cell lymphoma		Temsirolimus (mTORC1 inhibitor)	NCT01078142	I
Multiple myeloma		Everolimus + Panobinostat (HDAC inhibitor)	NCT00918333	I/II
Multiple myeloma, Lymphoma		Everolimus + Sorafenib (multikinase inhibitor)	NCT00474929	I/II
Acute monoblastic leukaemia		Lithium	NCT01820624	I
Advanced haematological malignancies		Triciribine	NCT00642031	I
Myelodysplasic syndrome and		Autophagy inducer + Azacitidine	NCT01210274	Recruiting
Acute myelogenous leukaemia				
Relapsed/Refractory NHL or HL		CAL-101	NCT01306643/NCT01393106	I/II
			NCT01282424	
Relapsed/Refractory NHL		CAL-101 + Rituximab +/− Bendamustine	NCT01088048/NCT01732913	I
			NCT01732926	
		CAL-101 + GS-9973 (Syk inhibitor)	NCT01796470	II
Lymphoma malignancies		IPI-145 (PI3Kd and PI3Kg inhibitor)	NCT01476657	I
		CC-223 (dual mTORC1 and mTORC2 inhibitor)	NCT01177397	I/II
Relapsed/Refractory/Newly diagnosed lymphoma		Everolimus in combination therapies (+/− Antibodies +/− TKIs +/− Chemotherapy)	NCT00869999/NCT01334502	I/II
			NCT01198665/NCT01665768	
			NCT01854606/NCT01341834	
			NCT01075321/NCT01567475	
			NCT00352443/NCT01453504	
Relapsed/Refractory lymphoma		Everolimus	NCT00436618	II
		Everolimus + Panobinostat	NCT00962507/NCT00978432	I/II
		Ridaforolimus (mTORC1 inhibitor)	NCT00060632/NCT00060645 NCT00086125	I/II
		Ridaforolimus + Vorinostat (HDAC inhibitor)	NCT 01169532	I/II
